# Fluorescence and Time-Delayed Lasing during Single Laser Pulse Excitation of a Pendant mm-Sized Dye Droplet

**DOI:** 10.3390/molecules24244464

**Published:** 2019-12-05

**Authors:** Mihai Boni, Ionut Relu Andrei, Mihail Lucian Pascu, Angela Staicu

**Affiliations:** 1 National Institute for Laser, Plasma and Radiation Physics, 077125 Magurele, Ilfov, Romania; mihai.boni@inflpr.ro (M.B.); ionut.andrei@inflpr.ro (I.R.A.); mihai.pascu@inflpr.ro (M.L.P.); 2 Faculty of Physics, University of Bucharest, 077125 Magurele, Ilfov, Romania

**Keywords:** laser-induced fluorescence, lasing, laser dye, mm-sized droplets, optical signal sampling

## Abstract

Fluorescence and lasing emission that are produced separately in time during excitation laser pulse for an mm-sized Rhodamine 6G dye-water droplet are reported. The droplet acts as a quasi-spherical closed optical resonator and due to multiple internal reflections, the resonant amplified emission is delayed with respect to fluorescence emission. Measurements of the temporal evolution of the droplet’s emission were performed by varying the signal acquisition gate width and gate delay with respect to the pumping pulse. The droplet emission spectra are structured in two bands which appear one after the other in time: first, the fluorescence emission band which follows pumping laser pulse time shape and then a second band, the lasing band, placed at shorter wavelengths and formed in time after the peak of the pumping laser pulse intensity, on the pulse tail. The lasing threshold pumping intensity is much lower than those for typical dye lasers.

## 1. Introduction

Droplets are a subject of increased interest, particularly from the point of view of their microfluidic and optical properties. They were already studied in different conditions and positions, such as sessile on a hydrophobic material [1], pending to a needle [2,3] or to an optical fiber, free falling, levitating in acoustic [4,5], magnetic [6], or electromagnetic [6,7] fields, manipulated by light (optical tweezer) [8], immersed in an immiscible liquid [7,9], or traveling through capillaries in the so-called lab-on-a-chip systems [10,11,12].

Optical phenomena specific to droplets always represented an attractive field for basic research and for applications in biology [13,14] or chemical sensing [15,16]. Due to their quasi-spherical shape and to the ratio between the refractive index of droplet’s material and the surrounding medium, electromagnetic waves may be trapped in the sphere by multiple total internal reflections which lead to optical resonances described as whispering gallery modes (WGM) and reported typically for droplets of hundreds of nanometers in diameter [4,17]. Droplets at volumes lower than or equal to 1 µL have an almost perfect spherical shape and may act as a natural optical cavity. When optically pumped, they emit fluorescence radiation similar to a higher volume bulk sample (mL), but may also emit lasing radiation that makes them, at the limit, micro-lasers [2].

The emission spectra of a pumping laser–droplet system may be tuned by changing the size [7], shape, composition, and temperature of droplets constituted in liquid micro cavities, as well as by the pumping laser energies and geometry of excitation and collection of fluorescence radiation [3].

In our laboratory, we started an extensive study of the emission of mm-sized pendant droplets containing Rhodamine 6G (Rh6G) dye solution [3,18] and dye emulsions [2]. In previous reports [2,3], we analyzed the emission of larger droplets (7 µL or 10 µL) of Rh6G solutions excited at lower power density with respect to the pumping lasing threshold. Also, we analyzed the geometry of excitation and collection of emitted radiation [3], and the effect of emulsion particles on the droplet emission [2]. In [18], we studied the evolution from pulse to pulse of the emission of a 1-µL Rh6G droplet when excited with a sequence of laser pulses.

In this paper, the temporal behavior of the emitted radiation collected from a single pendant droplet of 1 µL volume and containing Rh6G in distilled water at several concentrations is studied, when pumped with a 532-nm single pulsed laser beam. The emission was characterized by varying the spectrograph signal acquisition gate time width and gate delay with respect to the excitation pulse. The droplet emission spectra were structured in two bands which appeared one after the other in time during the excitation pulse. The excitation beam produced first a fluorescence emission band which followed the pumping laser pulse time shape. The second band placed at shorter wavelengths was formed in time after the peak of the pumping laser pulse intensity, on the tail of the pulse. Therefore, the second emission band was built in the cavity and it was delayed with respect to the spontaneous emission that appeared together with the laser pulse. The results on the droplets were compared with the bulk ones obtained in conditions for which lasing effects appear [18].

To our best knowledge, the reported behavior was the first observation about the mm-sized droplets’ emission, spectrally structured in two bands, which were separated in time during the excitation pulse lifetime.

## 2. Results and Discussion

LIF measurements on 1-µL pendant droplets containing Rh6G water solutions at concentrations ranging between 10^−5^ M and 10^−3^ M were performed. Using temporal sampling, an experimental time deconvolution of droplet emission spectra for each concentration was obtained.

For each concentration, applied on the droplet was the maximum beam energy which did not distort the droplet shape. Above this energy, unresonant interaction effects (generated by light pressure) [19] took place and produced vibrations or even droplet destruction. In addition, by inducing small vibrations in the droplet, the spherical shape is distorted and the collected signal can be unpredictably altered.

**Figure 1:**
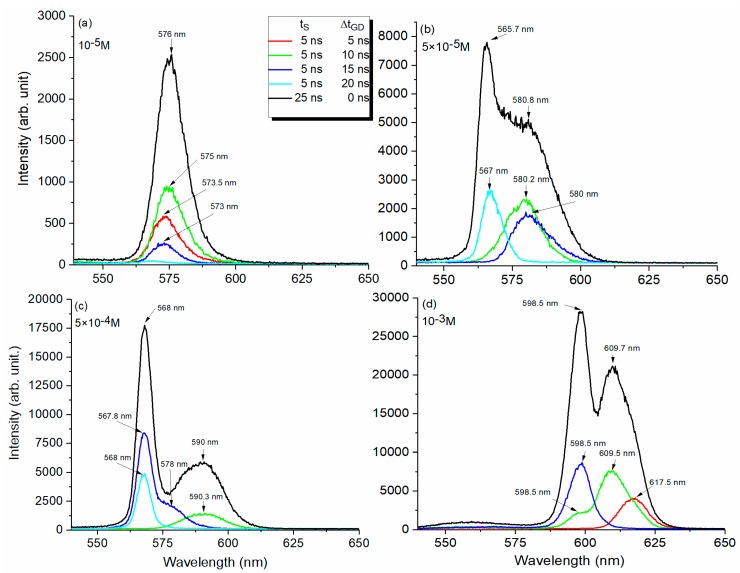
Emission spectra of a 1-µL droplet containing Rh6G water solutions at: (**a**) 10^−5^ M and excitation pulse energy 10 mJ; (**b**) 5 × 10^−5^ M at pulse energy 18 mJ; (**c**) 5 × 10^−4^ M at pulse energy 18 mJ; and (**d**) 10^−3^ M, at pulse energy 20 mJ. Sampling spectra obtained keeping t_s_ = 5 ns and modifying Δt_GD_, and the global spectra, using t_g_ = 25 ns and Δt_GD_ = 0 ns. Each spectrum is measured for a single droplet interacting with a single laser pulse.

Figure 1a–d synthesize the emission spectra obtained from 1-µL droplets at used concentrations. By recording the spectra with different gate widths (t_g_ and t_s_) and sampling gate delays (Δt_GD_) one can observe how emission radiation is formed in time.

For a 10^−5^ M Rh6G droplet (Figure 1a), emission spectra obtained at 10-mJ pumping energy showed one peak at about 576 nm and its shape did not change when the sampling gate delay was modified. This was in agreement with literature reports about the fluorescence emission of large-volume Rh6G dye-water droplets [3]. On the other hand, the peak intensity increased when the sampling delay time was increased, reaching a maximum for Δt_GD_ = 10 ns. Then, it showed a faster decrease that followed the pumping pulse intensity time distribution.

In contrast, all global measurements performed on droplets at 5 × 10^−5^ M, 5 × 10^−4^ M, and 10^−3^ M Rh6G concentrations in water showed a two-peak structure of the emission spectra (Figure 1b–d, respectively).

At 5 × 10^−5^ M and 18 mJ pumping energy (Figure 1b), for global gate width t_g_ = 25 ns and Δt_GD_ = 0 ns, two distinct and well-shaped peaks were observed: one at 580.8 nm and the other at 565.7 nm. By increasing the dye concentration from 10^−5^ M to 5 × 10^-5^ M, the right peak which was assigned to fluorescence emission was shifted to longer wavelengths, from 576 nm to 580.8 nm. Resolved emission spectra depicted in Figure 1b and obtained with a gate of 5 ns and different delays (Δt_GD_) show that no signal was obtained for the first two time intervals and the emitted signals for sampling intervals number 3 and 4 (gate delay 10 ns and 15 ns) contained only the fluorescence radiation (the right peak). If a gate delay of Δt_GD_ = 20 ns was applied, the right peak vanished and a second one rose up on the blue side at 567 nm (the left peak). Practically, the excitation beam produced first the fluorescence emission band and then a second peak was formed at the end of the laser excitation pulse. The second peak seemed to be built in the cavity and was delayed compared to the spontaneous emission, which appeared together with the laser pulse and had the maximum at the same moment in time.

Similar results on droplet emission were obtained for 5 × 10^-4^ M (Figure 1c) and 10^−3^ M (Figure 1d) concentrations, when pumping energies of 18 mJ and 20 mJ, respectively, were used. With the increase of dye concentration, both emission peaks showed a bathochromic shift. This was similar to the behavior of fluorescence emission maxima movement towards longer wavelengths when dye concentration increased in the case of bulk samples excitation and was due to the reabsorption/reemission processes and generation of non-fluorescent aggregates [20,21].

The wavelength of the delayed emission peak, left peak, corresponded to the maximum dye gain curve, had a resonant character and was associated with lasing emission [18].

By increasing the dye concentration, the fluorescence peak from 576 nm moved towards 610 nm and the lasing peak (observed starting with 5 × 10^−5^ M) shifted from 565.7 nm to 598.5 nm (Figure 1, global acquisition spectra).

When emission spectra were investigated by temporal sampling, the disappearance of the fluorescence peak and the rise of the lasing peak were produced faster with the concentration increase. In this sense, one may observe that for the 5 × 10^−4^ M concentration, the lasing peak appears for a delay gate of 15 ns compared to 10^−3^ M when the lasing appears after 10 ns.

It is worthwhile to mention that for all concentrations, the emission signals integrated over 25 ns appeared as an envelope of the two bands which were resolved for shorter integration time.

**Figure 2:**
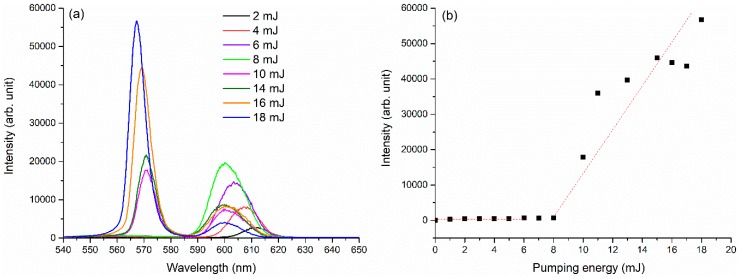
(**a**) Emission spectra measured for a 1-μL pendant droplet containing Rh6G solution in distilled water at 5 × 10^−4^ M versus pumping laser energy; (**b**) the lasing peak intensity versus pumping energy for a 1-μL pendant droplet containing Rh6G solution in distilled water at 5 × 10^−4^ M.

Emission spectra were also analyzed function of pumping laser energy. In Figure 2a, the emission spectra recorded for Δt_GD_ = 0 and temporal window 25 ns and 5 × 10^−4^ M dye concentration are shown as function of the excitation energy which varied from 2 mJ to 18 mJ. At energies lower than 10 mJ, the emission spectrum had only one peak which moved from 611 nm to 599 nm with the pumping energy increase. When pumping pulse energy was about 10 mJ, the second peak placed to the left appeared at 571 nm.

Considering the dependence of the spectra on pumping beam energy and on delay time, one may conclude that the left peak has a threshold energy at about 10 mJ and the delay time for its appearance is 10 ns to 15 ns measured from laser pulse start. This suggests that the peak belongs to a lasing emission, probably due to enhancement of that emitted light which is trapped in a droplet resonant cavity. On the other hand, the droplet emission behavior can be, as well, an effect of stimulated scattering which may contribute to the power density distribution of the optical field within the droplet.

The variation of left peak intensity with the pumping energy is presented in Figure 2b. It can be observed that this emission band has typical intensity behavior as a lasing radiation with a clear show-up threshold. The pumping laser intensity corresponding to 10 mJ threshold energy is 8 W/cm^2^. This is much lower than the value for typical dye lasers of 10 kW/cm^2^ [21].

Comparing these results with previous ones obtained on larger droplets of 7 µL or 10 µL containing R6hG water solutions [2,3] where lower pumping power densities were applied, we concluded that the threshold of lasing appears to be dependent on droplet size and the pumping energy trapped in the droplet resonant cavity. Furthermore, the addition of strong scattering media in the droplet such as in emulsions [2] decrease the threshold of lasing. Even though we had 10 mJ applied on a 7-µL droplet, due to the presence of emulsion small particles that act as light scatterers, we could observe the lasing band compared to the simple dye solution in the same conditions.

**Figure 3:**
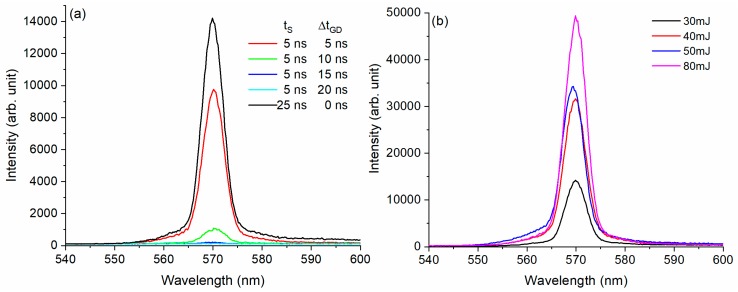
The emission spectra obtained for a bulk sample of Rh6G in distilled water with a concentration 5 × 10^−4^ M at (**a**) pumping energy 20 mJ, temporal gate width 5 ns, gate delay between 0–20 ns; (**b**) pumping energy between 30 mJ and 80 mJ, temporal window 25 ns and Δt_GD_ = 0. Each spectrum is measured on a single laser pulse.

For comparison, the same types of measurements were performed on bulk samples placed in a conventional spectrophotometric cuvette with 1 cm optical path length. The results obtained for a Rh6G aqueous solution at 5 × 10^−4^ M are shown in Figure 3a,b. Compared with other reports on the fluorescence spectra of Rh6G measured in bulk at low pumping energies [3], here a narrow emission peak is recorded. This effect is known to appear at high pumping energies, where the lateral windows of the cuvette play the role of a resonant cavity [21].

The dependence of the emission signal on detection gate delay (Δt_GD_) (Figure 3a) and on pumping energy (Figure 3b) was investigated as in the droplet case. In contrast to the droplet’s emission, the temporal sampling of the signal had no effect and the intensity of the signal followed the temporal shape of the pumping pulse. In addition, the increase of pumping energy did not affect the behavior and the shape of the emission spectra of the bulk sample; only one peak was observed and a second peak did not show up.

Therefore, one may conclude that the droplet’s spherical shape, correlated with its small dimensions, determines the characteristics of the emission spectra of pulsed laser-pumped mm-sized dye-doped droplets. A lasing band placed at a shorter wavelength with respect to the fluorescence band is built up in the droplet which acts as a spherical cavity. For a droplet, the lasing beam has much lower threshold pumping energy and is formed later in time compared to fluorescence radiation.

## 3. Materials and Methods

**Figure 4:**
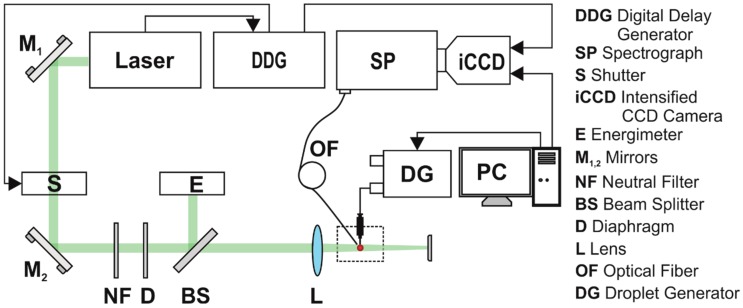
Experimental set-up for laser induced fluorescence measurements.

In Figure 4 is shown the experimental set-up used for laser-induced fluorescence (LIF) measurements on pendant droplets, described in detail in previous reports [2,3,18]. The 1-µL droplets were produced in air by a droplet generator (Microlab 560C, Hamilton, Reno, NV, USA) through a capillary tip and pumped at 532 nm with a single laser pulse. The droplets contained solutions of Rh6G dye in distilled water at concentrations between 10^−5^ M and 10^−3^ M. Measurements were made using a fresh droplet for each pumping pulse/spectra acquisition.

In order to pump the pendant droplet, the laser beam was sent on it into a slightly convergent geometry (beam divergence was around 4.83°), processed with a lens with focal length f = 150 mm. The 1-µL droplet was generated and placed at 122 mm with respect to lens L in such a way that the laser beam spot fully covered it.

The beam waist in the interaction plane had the linear dimension around the droplet’s diameter (1.24 mm). Since the laser beam is not focused on the droplet, one may use a higher beam energy which does not destroy it, so that in total, a more intense fluorescence emission is produced [14,18,19,22].

**Figure 5:**
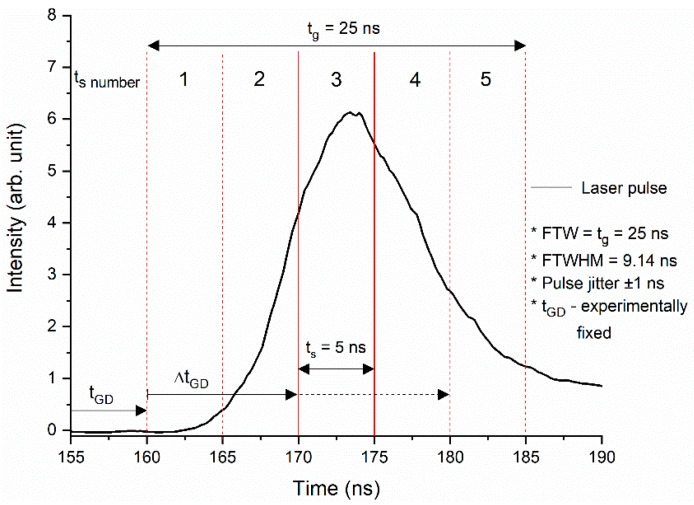
Temporal shape of the laser pulse. The time intervals indicate: global acquisition gate width t_g_ = 25 ns, temporal sampling acquisition gate width t_s_ = 5 ns, and sampling gate delay time Δt_GD_ (variable from 0 ns to 20 ns). Vertical solid red lines mark the current sampling gate width; dashed red lines mark the previous and following sampling gates. The horizontal (dashed) black arrow marks Δt_GD_ gate delay progressively increased in steps of 5 ns.

The laser source was a pulsed Nd:YAG laser (Surelite II, Continuum, San Jose, CA, USA), frequency doubled, emitting laser radiation at 532 nm with 10 pps pulse repetition rate. The working energy of the laser was decreased using a low value for the Q-switch delay laser parameter. In these conditions, the laser pulse had 25-ns full time width (FTW) and the measured full time width at half maximum (FTWHM) was 9.14 ns (Figure 5). The experiments on droplets were carried out at pumping energies per pulse that varied between 10 mJ and 20 mJ. A set of neutral density filters were, in addition, used to adjust the energy. Droplet-emitted light was collected with an optical fibre (1-mm core diameter) perpendicularly placed to the excitation beam in the droplet’s immediate proximity (Figure 4).

Bulk measurements were performed using the same experimental set-up as in the droplet case by placing 1 mL dye solution in a spectrophotometric cuvette, by removing the lens, and by using higher pumping energies from 20 mJ/pulse to 80 mJ/pulse.

The emitted light was spectrally analysed using a spectrograph (SpectraPro 2750, Acton Research, Trenton, NJ, USA coupled with an intensified CCD camera (iCCD PIMAX 1024 RB, Princeton Instruments, Trenton, NJ, USA). Spectra were recorded with an optical resolution of 0.3 nm in the 500 nm–700 nm spectral range. The iCCD camera was synchronized with the laser using a digital delay generator (DDG) ) (DG 535, Stanford Research Systems, Sunnyvale, CA, USA).

The signal acquisition was made by two methods: global and temporal sampling. In Figure 5 is shown the laser pulse temporal shape. A time gate delay t_GD_ was experimentally fixed, being defined as the time interval at which the laser pulse reaches the droplet. This was established after preliminary checks, and it was introduced for the spectrograph’s gate opening with respect to a Time To Live (TTL) synchronization signal received from the DDG. Other parameters used were: t_g_—global acquisition gate width that equals the pulse FTW, 25 ns; t_s_—temporal sampling acquisition gate width, which is 5 ns, and numbered 1 to 5 corresponding to the number of samplings performed during the excitation pulse lifetime; Δt_GD_—the sampling gate delay introduced for the spectrograph’s gate opening and added to t_GD_.

Global measurements were performed keeping the spectrograph acquisition gate open t_g_ = 25 ns (i.e., the whole FTW pulse). In this case, no delay (Δt_GD_ = 0 ns) was applied to collect the LIF signal with respect to t_GD_, and the full Rh6G spectrum was collected. For temporal sampling, with t_s_ = 5 ns fixed, the gate was moved in steps of 5 ns (Δt_GD_ taken as a multiple of 5 ns), so that the sampling gate delays in Figure 5 are 0, 5, 10, 15, and 20 ns. The 25-ns laser pulse lifetime was scanned and up to 5 spectra were collected, each of them containing the signal integrated over 5 ns. Acquisition by temporal sampling made it possible to analyze the behavior of emitted light during the laser beam droplet interaction.

## 4. Conclusions

In this paper, it was demonstrated that by changing the geometry of the fluorescent medium from bulk to droplet, significant changes occurred in the emission spectra obtained during laser pulse excitation. The main difference was the appearance of a new emission peak, assigned to lasing emission, blueshifted and time-delayed with respect to the emitted fluorescence peak. The two emission peaks were analyzed as function of solution concentration and pumping energy, as well as by using global and temporal sampling acquisition procedures.

A new method was used to distinguish temporal changes of droplet emission by varying the signal acquisition gate width and/or the gate delay with respect to the laser excitation pulse. It was highlighted how the two peaks evolved during excitation pulse lifetime: first, a fluorescence emission band was formed and followed the laser pulse time shape, and secondly, a band placed at shorter wavelengths showed up after the peak of pumping laser pulse intensity, on the pulse tail. This second emission band was built in the cavity and it was delayed by the spontaneous emission, which appeared together with the laser pulse and had a maximum in the same time. The lasing beam had a much lower threshold of pumping energy compared to the conventional dye-active media. These results could make available laser sources emitting in a well-controlled way that have small dimensions and may find many applications.
